# Calcineurin inhibition rescues alloantigen-specific central memory T cell subsets that promote chronic GVHD. Reply.

**DOI:** 10.1172/JCI184869

**Published:** 2024-10-15

**Authors:** Ping Zhang, Simone A. Minnie, Geoffrey R. Hill

**Affiliations:** 1 Fred Hutchinson Cancer Center, Seattle, Washington, USA.; 2 QIMR Berghofer Medical Research Institute, Brisbane, Queensland, Australia.

**Keywords:** Transplantation, Bone marrow transplantation, T cell development

**The authors reply:** Senjo et al. recently transplanted equal and high numbers of congenic CD8^+^ 2C T cell receptor–transgenic (CD8^+^ 2C TCR–Tg) and polyclonal B6 (CD4^+^ and CD8^+^) T cells into lethally irradiated B6D2F1 mice and determined T cell differentiation in the presence/absence of cyclosporin, dosed orally at 25 mg/kg/day for 14 days (1). Absorption was not validated by drug levels. Interpretation was heavily influenced by single-cell RNA-seq (scRNA-seq) analyses on day 14 with subsequent protein analyses, performed principally on CD8^+^ 2C TCR–Tg T cells. In contrast, our findings were primarily in alloreactive CD4^+^ T cells, with validation of expansion kinetics using small numbers of H-Y–specific CD4^+^ TCR–Tg T cells (2). We noted preferential CD4^+^ central memory T cell (Tcm) generation in both polyclonal and HY-TCR–Tg T cells in the presence of a calcineurin inhibitor (CNI) and importantly, this expansion was dose and agent (tacrolimus > cyclosporin) dependent (2).

Senjo et al. concluded from their scRNA-seq data that cyclosporin promoted the expansion of transitory-exhausted (transitory-Tex) CD8^+^ T cells based on reduced PD-1, TIGIT, and TOX, and increased CX3CR1 RNA expression relative to other T cell clusters. We note that the population expanded by cyclosporin in cluster C1 contains a large fraction of TOX^–^ cells, as acknowledged by Senjo et al. in their recent Letter to the Editor (3). Consistent with this, the protein data in Senjo et al. (Figure 3F) (1) demonstrate that approximately 50% of 2C TCR-Tg T cells are TOX^–^ with cyclosporin. They did not analyze memory or exhausted T cell subsets by flow cytometry. Of note, CX3CR1 expression is consistent with effector/memory T cells and does not delineate transitory-Tex in the absence of TOX expression (4).

Senjo et al. assert that Ly6C specifically marks transitory-Tex based on scRNA-seq data (Figure 1E and ref. 3). Ly6C is broadly expressed on memory CD8^+^ T cells (5) and is most highly expressed on antigen-induced Tcm subsets (6). Indeed, Figure 3I of the Senjo et al. paper (1) shows that 64% of T cells expressed Ly6C in the presence of a CNI, but a large fraction of Ly6C^+^ cells appear TOX^–^. Thus, Ly6C does not discriminate transitory-Tex cells from other CD8^+^ T cell subsets after bone marrow transplantation (BMT) with a CNI and in fact, Ly6C^+^CD8^+^ T cells are enriched for nonexhausted (TOX^–^) T cells (Figure 1). Thus, the flow cytometry data in Figure 3, F and I of Senjo et al. and our own align. Critically, Senjo et al. note that they transferred Ly6C^+^ T cells (including CD4^+^ T cells), which gave rise to chronic GVHD (cGVHD) and the generation of TOX^+^ exhausted and TOX^–^ effectors. The latter confirms that these cells are not a pure population of transitory-Tex, given that this population is irreversibly committed to exhaustion (i.e., should not generate TOX^–^ effector T cells; ref. 7). Conversely, we transferred purified populations of CNI-induced CD4^+^ Tcm, which preferentially generated cGVHD.

We acknowledge that most of our analyses were on day 7 or 17 after BMT, the latter 4 days after withdrawal of the CNI. Nevertheless, we undertook analyses at the same day 14 time point (Figure 6 and ref. 2) in the same model (but without 2C TCR-Tg T cells) as Senjo et al. We demonstrated that in the presence of relevant systemic CNI levels (confirmed by drug levels), the expression of TOX and PD-1 was profoundly inhibited in CD4^+^ and CD8^+^ T cells, and all stages of T cell exhaustion were suppressed. Conversely, proportions of effector/memory subsets were expanded.

In conclusion, interpretation of established T cell subsets in Senjo et al. is difficult in the absence of CNI levels and validation of scRNA-seq analyses by flow cytometry. In our hands, CNIs, and particularly tacrolimus, profoundly impaired all stages of T cell exhaustion after BMT and instead promoted alloreactive CD4^+^ Tcm that generated cGVHD. The data in Senjo et al. are consistent with this, although we postulate that CNI levels were significantly lower in their studies. Other than Senjo et al. defining Ly6C^+^TOX^–^ T cells as “exhausted” rather than effector/memory and their focus on CD8^+^ rather than CD4^+^ T cells, the findings in both papers are concordant (i.e., that CNIs rescue alloreactive T cell clones that promote cGVHD).

## Figures and Tables

**Figure 1:**
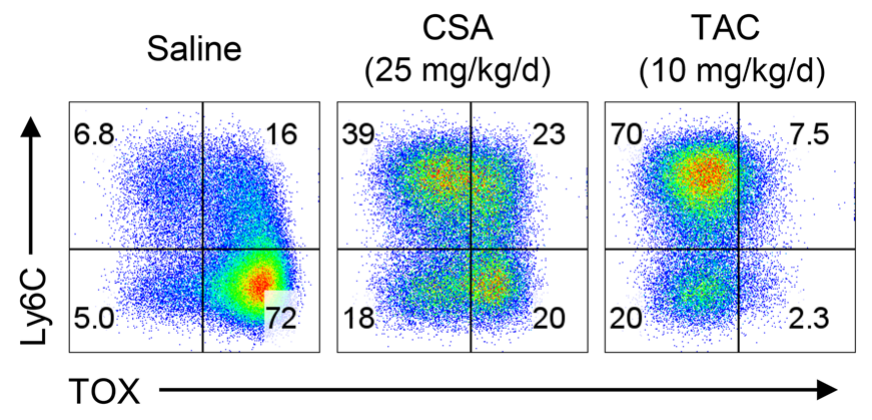
B6D2F1 recipients of B6 BM plus T cells were treated with saline, cyclosporin (CSA), or tacrolimus (TAC) from day 0 to 13. Expression of TOX and Ly6C in splenic CD8^+^ T cells on day 14 (representative from 2 experiments).

## Version 1 10/15/2024

Electronic publication
